# Joint segmentation and classification of hepatic lesions in ultrasound images using deep learning

**DOI:** 10.1007/s00330-021-07850-9

**Published:** 2021-04-21

**Authors:** Hwaseong Ryu, Seung Yeon Shin, Jae Young Lee, Kyoung Mu Lee, Hyo-jin Kang, Jonghyon Yi

**Affiliations:** 1 Department of Radiology, Pusan National University Yangsan Hospital, Yangsan, Republic of Korea; 2 National Institutes of Health, Bethesda, MD 20892 USA; 3 Department of Radiology and the Institute of Radiation Medicine, Seoul National University Hospital, Seoul, Republic of Korea; 4 Department of Radiology, Seoul National University College of Medicine, Seoul, Republic of Korea; 5 Department of Radiology, Seoul National University Hospital, 101 Daehak-ro, Jongno-gu, Seoul, 03080 Republic of Korea; 6 Department of Electrical and Computer Engineering, Seoul National University, 1, Gwanak-ro, Gwanak-gu, Seoul, 08826 Republic of Korea; 7 Medical Imaging R&D Group, Health & Medical Equipment Business, Samsung Electronics Co., Ltd., Seoul, Republic of Korea

**Keywords:** Deep learning, Ultrasonography, Liver

## Abstract

**Objectives:**

To develop a convolutional neural network system to jointly segment and classify a hepatic lesion selected by user clicks in ultrasound images.

**Methods:**

In total, 4309 anonymized ultrasound images of 3873 patients with hepatic cyst (*n* = 1214), hemangioma (*n* = 1220), metastasis (*n* = 1001), or hepatocellular carcinoma (HCC) (*n* = 874) were collected and annotated. The images were divided into 3909 training and 400 test images. Our network is composed of one shared encoder and two inference branches used for segmentation and classification and takes the concatenation of an input image and two Euclidean distance maps of foreground and background clicks provided by a user as input. The performance of hepatic lesion segmentation was evaluated based on the Jaccard index (JI), and the performance of classification was based on accuracy, sensitivity, specificity, and the area under the receiver operating characteristic curve (AUROC).

**Results:**

We achieved performance improvements by jointly conducting segmentation and classification. In the segmentation only system, the mean JI was 68.5%. In the classification only system, the accuracy of classifying four types of hepatic lesions was 79.8%. The mean JI and classification accuracy were 68.5% and 82.2%, respectively, for the proposed joint system. The optimal sensitivity and specificity and the AUROC of classifying benign and malignant hepatic lesions of the joint system were 95.0%, 86.0%, and 0.970, respectively. The respective sensitivity, specificity, and the AUROC for classifying four hepatic lesions of the joint system were 86.7%, 89.7%, and 0.947.

**Conclusions:**

The proposed joint system exhibited fair performance compared to segmentation only and classification only systems.

**Key Points:**

*• The joint segmentation and classification system using deep learning accurately segmented and classified hepatic lesions selected by user clicks in US examination.*

*• The joint segmentation and classification system for hepatic lesions in US images exhibited higher performance than segmentation only and classification only systems.*

*• The joint segmentation and classification system could assist radiologists with minimal experience in US imaging by characterizing hepatic lesions.*

**Supplementary Information:**

The online version contains supplementary material available at 10.1007/s00330-021-07850-9.

## Introduction

Ultrasound (US) is a widespread first-line imaging modality used in the diagnosis of liver diseases given its low cost, nonionizing characteristics, portable features, and ability for real-time image acquisition and display. Many focal liver lesions are detected incidentally during the first evaluation or follow-up for a primary neoplasm or during surveillance for chronic liver diseases and cirrhosis; however, characterizing the incidental focal hepatic lesions by US imaging is challenging and occasionally shows low sensitivity in the detection of solid lesions due to low contrast between the lesion and the surrounding liver. In addition, large interobserver variability is noted based on the level of operator experience. The confusion created by overlapping US features of hepatic focal lesions is also a factor that limits interpretation [1–3]. To overcome these limitations, many computer-aided systems for hepatic lesion segmentation and classification, including deep learning, have been developed [4].

Fully automatic segmentation of existing tumors in US images has been considered difficult due to US imaging limitations, such as speckle noise, low contrast between tumors and surrounding tissues, and varied morphology and echogenicity according to scan direction [5]. In 2016, Xu et al [6] developed a semiautomatic segmentation system based on deep learning, which requires user clicks to segment a specific object in a given image. Despite its original target domain of natural images, this system is also adaptable to clinical images. Deep learning–based systems for lesion classification in US images have also been thoroughly investigated [7–12]. Most of the previous approaches take the region-of-interest (ROI), which is manually drawn by a radiologist, as input; however, manual segmentation is tedious and time-consuming, which limits usability in clinical practice [4, 5]. A possible solution involves cascading the segmentation and classification systems, where an ROI automatically extracted by the segmentation system is fed into the classification system. Although the segmentation and classification tasks can be performed separately, they may be related to each other and thus produce mutual benefits when performed simultaneously. Specifically, the segmentation could provide an ROI for classification, and the classification could provide any cues, such as desired shapes based on the lesion types, for segmentation. Therefore, the purpose of this study is to develop a convolutional neural network (CNN) system to jointly segment and classify a hepatic lesion selected by user clicks in US examination.

## Materials and methods

This study was performed with approval from our Institutional Review Board. The requirement for informed consent was waived given the retrospective nature of the data analysis and the use of fully anonymized US images.

### Dataset development

**Fig. 1:**
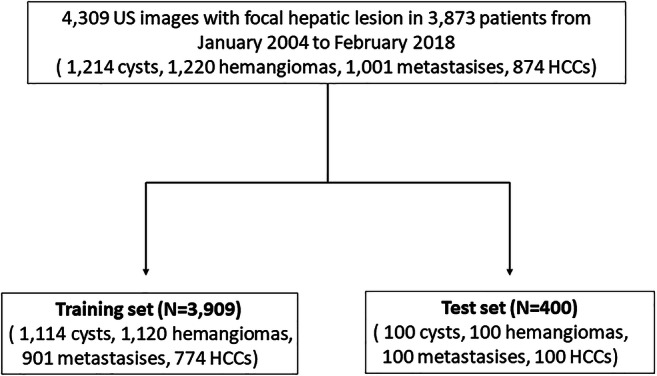
Study design. Note. HCC, hepatocellular carcinoma

For developing the dataset, we used 4309 US images with focal hepatic lesions from 3873 patients (1993 men, 1880 women; mean age, 61.0 years ± 11.6; age range, 14–94 years). US examinations were performed between January 2004 and February 2018 at a single institution. In terms of the lesion types contained in the images, 1214 images with hepatic cysts (mean size 16.8 ± 14.9 mm), 1220 images with hemangioma (mean size 17.4 ± 14.5 mm), 1001 images with metastasis (mean size 26.2 ± 15.7 mm), and 874 images with HCC (mean size 23.3 ± 14.7 mm) were included in this study. US data of benign lesions, such as hepatic cysts and hemangiomas, were obtained from patients who were referred to the Department of Radiology at our institution for screening for CLD or other diffuse liver diseases. These cysts and hemangiomas were diagnosed by typical findings in follow-up CT and MRI images. US data of malignant lesions, including metastasis and HCC, were obtained from patients who underwent liver resection or percutaneous liver biopsy for focal hepatic lesions performed within 6 months after US examination. All US images of the focal hepatic lesions regardless of the scanning plane (such as subcostal or intercoastal scan) were collected as grayscale images on the picture archiving and communication system. One representative image with maximum size as measured in the longest dimension of each focal hepatic lesion was used. Manual segmentation of the hepatic lesions was performed by two radiologists (J.Y.L. with 24 years of clinical experience in abdominal US, and H.W.R. with 5 years of clinical experience in abdominal US). These images were used as the reference standard for the lesion segmentation task. For each lesion type, the images were sorted according to the lesion area and divided into 10 groups of equal size (Appendix 1). To include lesions of various sizes in the test set, 10 images were randomly selected from each group for every lesion type, which resulted in 400 images in total. The training set was composed of the remaining 3909 images (Fig. 1). As a constraint, the images of one patient could not be in different sets.

### US examinations

US examinations were performed using Siemens Acuson Sequoia, S2000, and S3000 (Siemens AG); Samsung RS 80A ultrasound system (Samsung Medison); LOGIQ E9 ultrasound system (GE Healthcare); and iU22 or EPIQ (Philips Medical systems). The patients underwent US examination after fasting for at least 6 h. Conventional B-mode sonography using a convex probe was performed.

### Developing a joint segmentation and classification system

**Fig. 2:**
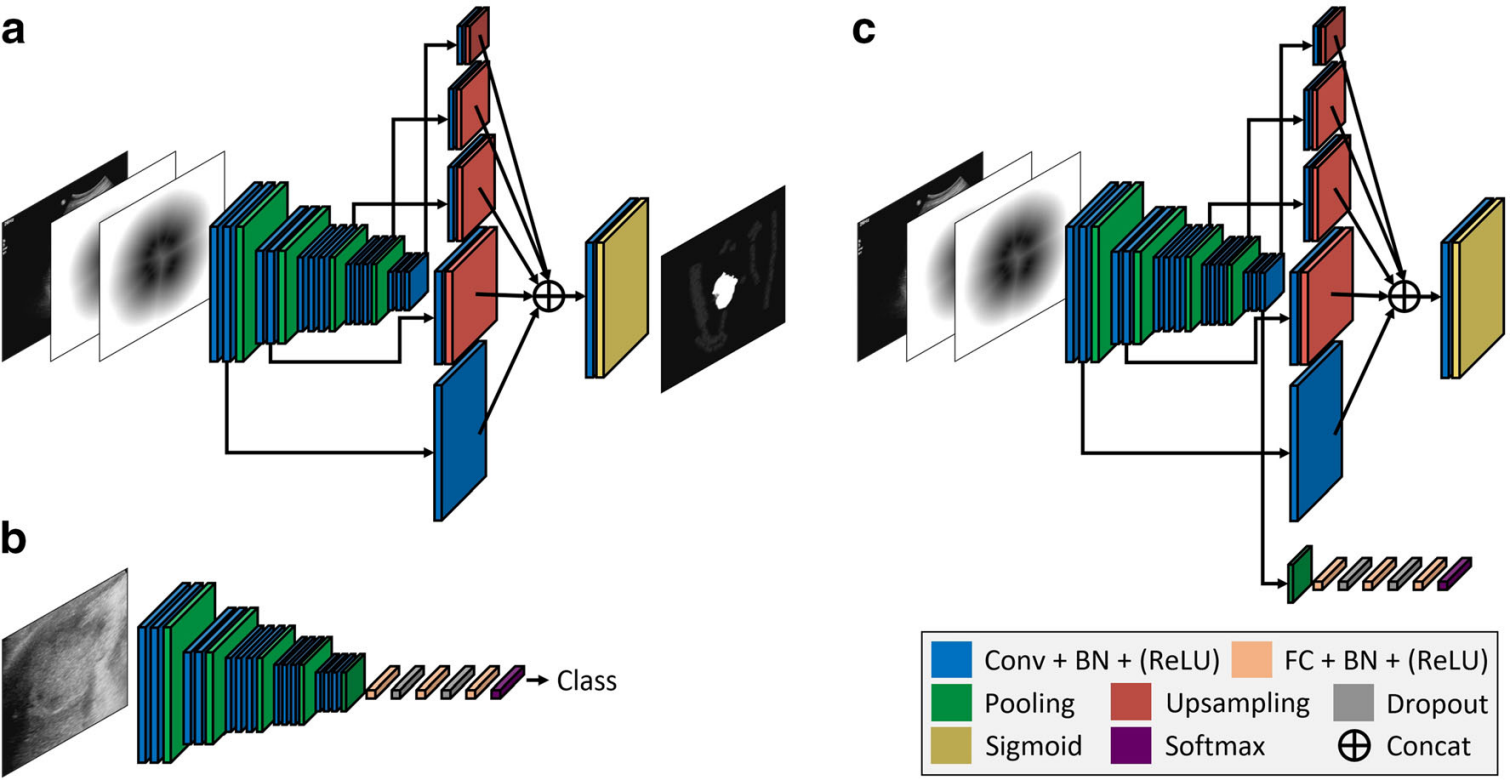
Network architecture for the proposed method. **a** The segmentation only system, which takes a three-channel image composed of a grayscale input image and two Euclidean distance maps of foreground and background clicks provided by a user as input. **b** The classification only system, which takes a local image patch surrounding a lesion of interest as input. **c** The joint segmentation and classification system, which takes the same input of the segmentation system and produces segmentation and classification predictions simultaneously. Conv, convolution layer; BN, batch normalization; ReLU, rectified linear unit; FC, fully connected layer; Concat, concatenation

**Fig. 3:**
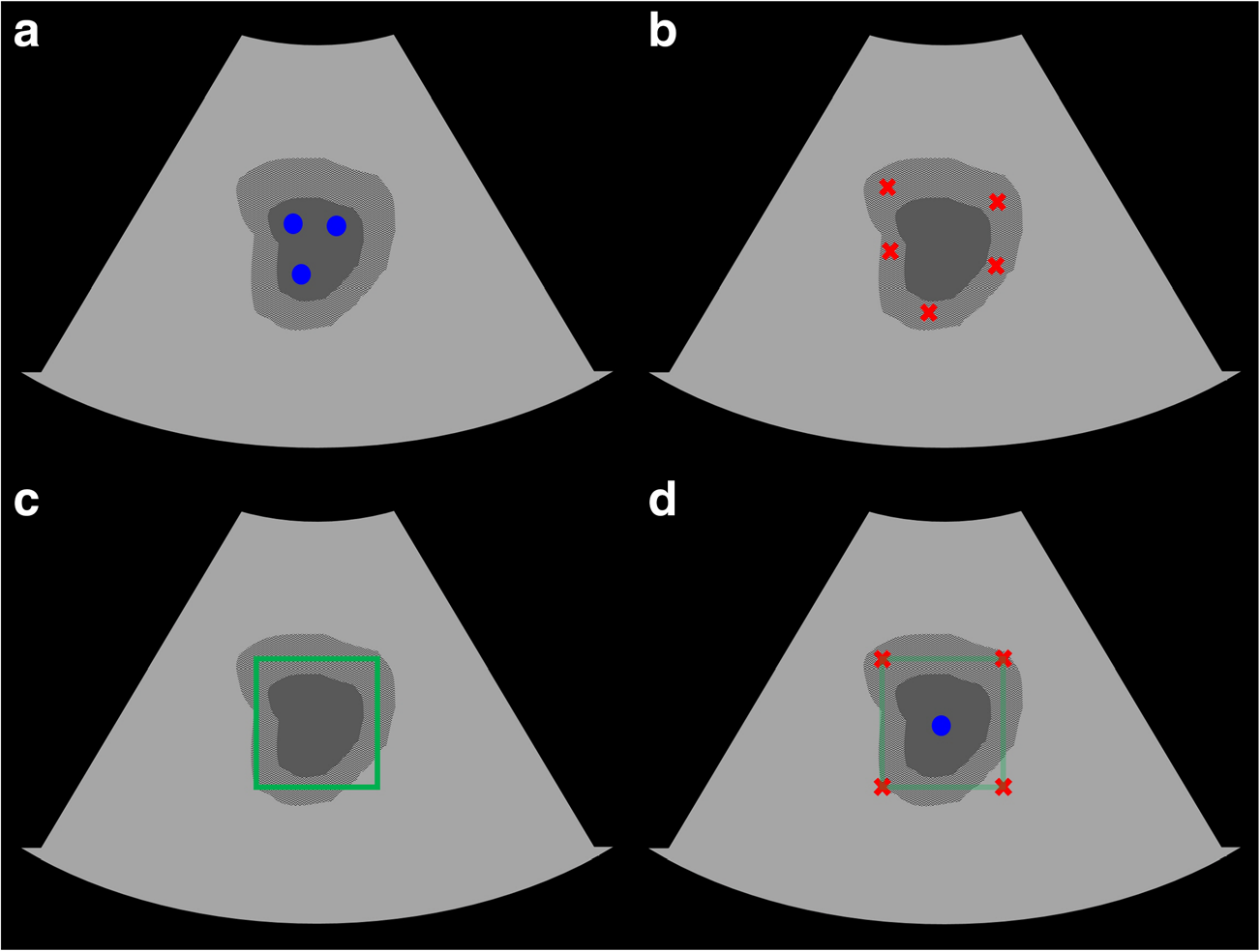
Schematic description of user inputs. The dark gray region represents a lesion of interest, and the shaded region is a set of background pixels that are within a certain distance range to the lesion. **a** An example of foreground user clicks (blue dots). **b** An example of background user clicks (red crosses). **c** An example of bounding box user input (green rectangle), which can be used for testing. **d** Transformation from the bounding box (**c**) to user clicks, including one foreground and four background clicks

We adapted a semiautomatic segmentation method proposed in [6] for hepatic lesion segmentation. The method takes the concatenation of an input image and two Euclidean distance maps of foreground and background user clicks as inputs and a foreground probability map as shown in Fig. 2a as the output. Therefore, we needed to obtain foreground and background clicks for each image to train the system. Because obtaining these clicks from real users is difficult, we instead used simulated user inputs that are automatically generated by following several rules. For foreground clicks, we randomly selected 1–5 pixels within a lesion (Fig. 3a). For background clicks, 0–10 pixels are randomly selected from the background pixels which are within a certain distance range to the lesion (Fig. 3b). Zero pixels are recorded when a user does not provide any background clicks. Using this method, we generated 15 click sequences for each foreground and background per image. Euclidean distance maps, which represent the minimum Euclidean distance of each pixel to a given set of user clicks, are computed and used as the actual input for the network. An image filled with 255 pixels is used as the background Euclidean distance map if no background click is provided**.**

We used a network architecture (Fig. 2a) adapted from that reported in [13] instead of the fully convolutional networks (FCNs) [14] that were originally used in [6]. The architecture uses bilinear interpolations instead of deconvolution layers for upsampling and has a smaller number of learnable parameters compared to the FCNs, which implies that our network is less prone to overfitting. We added batch normalization layers [15] to our network to further stabilize the network training. The proposed joint segmentation and classification system is implemented by augmenting the segmentation network with an additional classification branch (Fig. 2c). The classification branch, which is composed of several fully connected layers, predicts the lesion type selected by user clicks from the features of the shared convolutional layers. We also implemented the classification only system (Fig. 2b) to compare the performance to that of the joint system to demonstrate any benefit. The system is based on the VGG-16 network [16] with batch normalization [15] and has a structure when the segmentation branch is eliminated from the joint system. We used ideal local image patches centered on a lesion, which are twice the size of the lesion, during training and testing. We used the pixelwise focal loss and α-balanced focal loss for training the segmentation and classification systems, respectively [17]. The sum of two losses was used for the joint system. To train the joint network, we used a stochastic gradient descent optimizer with 0.9 momentum and a weight decay of 5 × 10^−4^. During the training of 150,000 iterations, the learning rates of 5 × 10^−4^ and 5 × 10^−5^ were used for the first and second halves, respectively. For data augmentation, we used horizontal image flipping, image scaling, and random brightness/contrast adjustment. The same hyperparameters were used when training the segmentation only network. For training the classification only network, the learning rates of 10^−3^ and 10^−4^ were used for the first and second halves of the whole iterations of 40,000, respectively. For better generalization, the early convolutional layers of all the networks were initialized using the other network trained with breast US images. Because the trained joint system operates with point-type user input, such as clicks, we could have diverse modes in testing. For example, a user scribble can be translated into a set of clicks that could be used for testing. In this study, we used two clinically useful test modes, which are separately based on one click and two clicks. In the one-click mode, a user selects only one pixel within a lesion. On the other hand, a user draws a bounding box surrounding a lesion by placing the top-left and bottom-right corners of it in the two-click mode (Fig. 3c). To test the two-click mode, we again generate five different bounding boxes for each test image by simulation. The top-left and bottom-right corners are sampled with a certain degree of positional error from the tight box of a lesion. The box is enlarged a little to ensure that it includes the lesion. Finally, the bounding box is transformed to five clicks, including one foreground click at the center of it and four background clicks at the corners, when it is used (Fig. 3d).

We used TensorFlow [18] to implement all the networks. The experiments were performed in an environment with Intel Core i7-7700K at 4.2-GHz CPU, 32-GB RAM, and Nvidia GeForce GTX 1080Ti (11 GB VRAM).

### Statistical analysis

The segmentation results of the segmentation only system and the joint segmentation and classification system were evaluated against the reference standard and assessed using the Jaccard index (JI) per image. The JI is calculated as the area of the intersection divided by the area of the union between the reference standard and segmentation result [19, 20]. While we present the performance of the two-click mode as our main result, we also provide the results of using a single click for comparison between the two modes. The classification performances of the classification only system and joint system were evaluated using accuracy and the area under the receiver operating characteristic curve (AUC). In terms of the classification, we conducted two experiments to predict benignity and malignancy and categorize the four types of hepatic lesions (hepatic cyst, hemangioma, metastasis, and HCC). We used five different user inputs for each test image to consider the different styles of user input.

## Results

### Segmentation performance

**Fig. 4:**
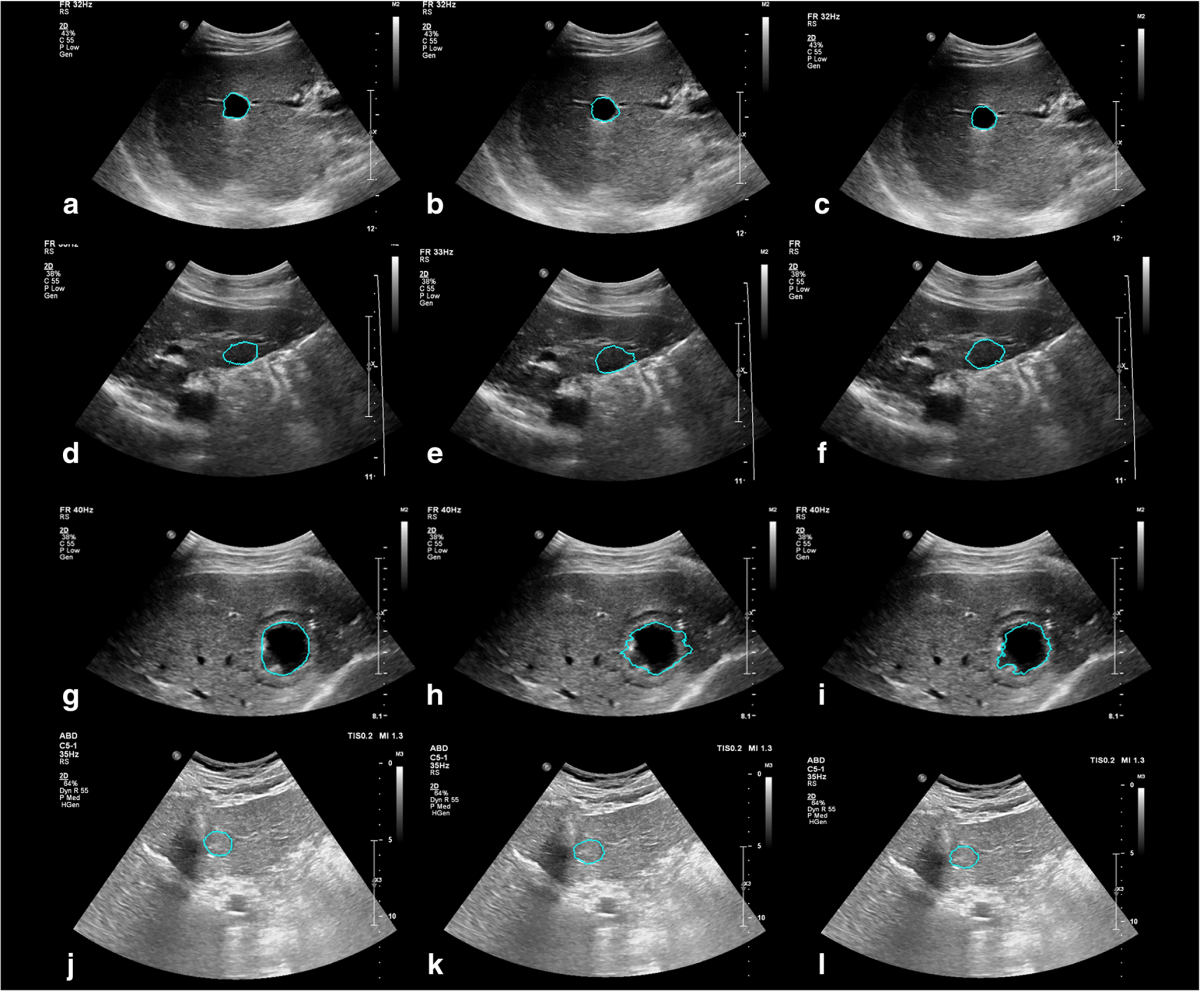
Examples of hepatic lesion segmentation using two clicks. *Left*: manual segmentation by a radiologist. *Middle*: result of the segmentation only system. *Right*: result of the joint segmentation and classification system. **a**–**c** cyst, **d**–**f** hemangioma, **g**–**i** metastasis, **j**–**l** hepatocellular carcinoma

**Fig. 5:**
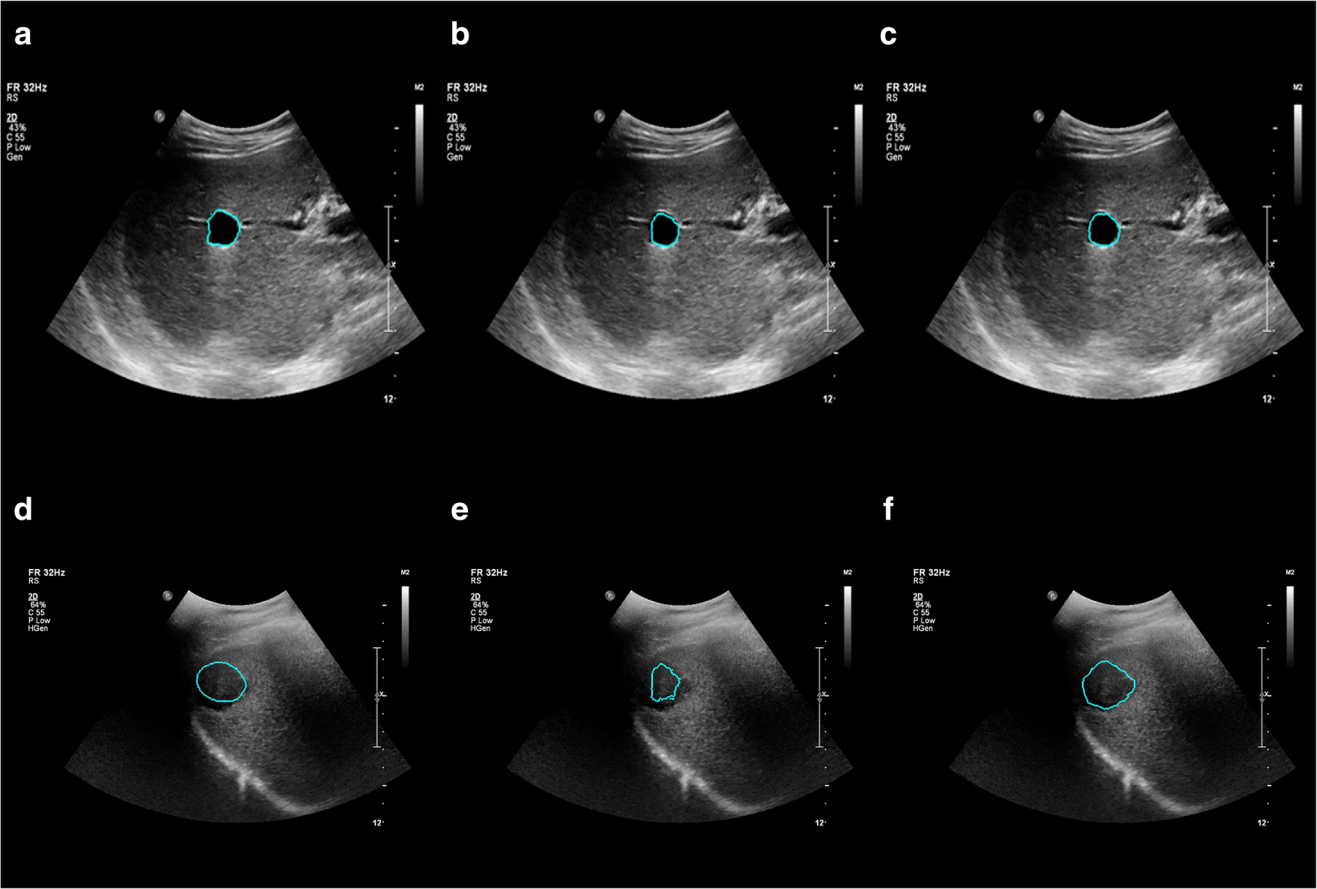
Examples of segmentation results of the joint segmentation and classification system using one or two clicks. *Left*: manual segmentation by a radiologist. *Middle*: result using one click. *Right*: result using two clicks (bounding box). **a**–**c** cyst, **d**–**f** HCC

We achieved a mean JI of 68.5% for the segmentation only system in the two-click mode. Compared with this, the joint segmentation and classification system achieved a mean JI of 70.0% when trained with the binary classification task (*p* < 0.001) and exhibited a comparable result (68.5%) when trained with the four-class classification task (*p =* 0.95) (Table 1). The comparable performance between the segmentation only system and the joint system was observed regardless of the size and type of hepatic lesions (Appendix 2). Nevertheless, the segmentation results of the joint system showed a tendency to better differentiate the lesion boundaries compared to the segmentation only system (Fig. 4). The mean JI of the joint system markedly decreased to 37.4% in the one-click mode. Although the system still produced accurate segmentations for lesions with clear margins and high contrast, it generated ambiguous and inaccurate boundaries for lesions with unclear margins, large size, and heterogeneous echogenicity (Fig. 5).

**Table 1:** Segmentation performance of the proposed systems

Systems	Training classification task	Segmentation (mean JI, %)	*p*
Segmentation only	-	68.5 ± 10.3	
Joint segmentation and classification	Benign/malignant	70.0 ± 10.9	< 0.001
	Cyst/hemangioma/metastasis/HCC	68.5 ± 12.2	0.95

Two-click user inputs were used for the segmentation only system and the joint system

Note. *JI*, Jaccard index; *HCC*, hepatocellular carcinoma

### Classification performance

**Fig. 6:**
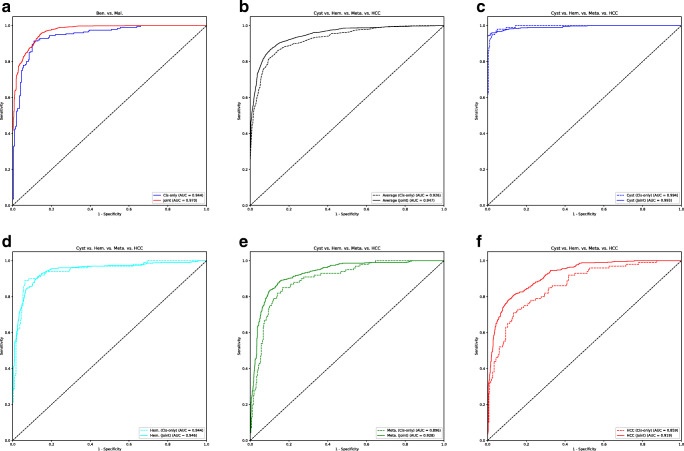
Receiver operating characteristic curves of the classification performance of the classification only system and the joint segmentation and classification system. Two-click user inputs were used for the joint system. **a** Classification between benign and malignant lesions. **b–f** Classification of the four types of hepatic lesions, including average (**b**), cyst (**c**), hemangioma (Hem.) (**d**), metastasis (Meta.) (**e**), and hepatocellular carcinoma (HCC) (**f**). AUC, area under the receiver operating characteristic curve; Cls-only, classification only system; Joint, joint segmentation and classification system

The classification accuracies of the classification only system were 89.8% and 79.8% for the binary and four-class classification tasks, respectively. Compared with this, the joint system achieved 90.4% (*p =* 0.48) and 82.2% (*p =* 0.01) for the respective tasks (Table 2). The joint system performed favorably against the classification only system across most of the lesion sizes. In terms of the lesion type, the largest increase of accuracy was observed in metastatic lesions (Appendix 3). In terms of ROC analysis, the AUC of the joint system for the binary class classification task was significantly higher than that of the classification only system (0.970, 0.944, *p* = 0.020). The AUC of the joint system for the four-class classification task was also significantly higher than that of the classification only system (0.947, 0.926, *p* = 0.040). For the binary classification task, the optimal sensitivity and specificity on the ROC curve of the joint system obtained by calculating Youden’s index [21] were 95.0% and 86.0%, respectively. For the four-class classification, the system yielded a sensitivity of 86.7% and a specificity of 89.7% (Fig. 6, Table 2). The confusion matrix of the four-class classification using the joint system showed that the accuracy for a cyst is the highest, whereas that of HCC is the lowest among all the lesion types (Table 3).

**Table 2:** Classification performance of the proposed systems

Classification task	Benign/malignant			Cyst/hemangioma/metastasis/HCC		
	Classification only	Joint segmentation and classification	*p*	Classification only	Joint segmentation and classification	*p*
AUROC	0.944 (0.919, 0.964)	0.970 (0.956, 0.982)	0.020	0.926 (0.905, 0.943)	0.947 (0.929, 0.963)	0.040
Sensitivity (%)	91.5 (87.0, 95.5)	95.0 (86.9, 98.1)	0.008	82.3 (79.7, 89.2)	86.7 (82.8, 91.4)	< 0.001
Specificity (%)	89.0 (84.5, 93.5)	86.0 (81.7, 94.1)	0.029	89.7 (83.0, 92.3)	89.7 (85.1, 93.3)	0.27
Accuracy (%)	89.8 (86.8, 92.8)	90.4 (87.6, 93.0)	0.48	79.8 (75.8, 83.5)	82.2 (78.7, 85.9)	0.01

Two-click user inputs were used

Note. Data are 95% confidence interval in parentheses. *HCC*, hepatocellular carcinoma; *AUROC*, area under the receiver operating characteristic curve

**Table 3:** Confusion matrix of hepatic lesion classification using the joint segmentation and classification system

	Results from the joint segmentation and classification system				Accuracy (%)
	Cyst	Hemangioma	Metastasis	HCC	
Cyst (*n*=500)	*472*	15	5	8	94.4
Hemangioma (*n*=500)	1	*413*	46	40	82.6
Metastasis (*n*=500)	0	46	*423*	31	84.6
HCC (*n*=500)	0	37	127	*336*	67.2
Total					82.2

Two-click user inputs were used.

Note. *HCC*, hepatocellular carcinoma

The mean execution time per image of the joint system was 73 ms with a NVIDIA GeForce GTX 1080Ti GPU card. The respective execution times for the segmentation only system and the classification only system were 73 ms and 10 ms. We note that an image patch should be cropped from a segmentation result before feeding into the classification system, which requires additional processing time, but is not required for the joint system.

## Discussion

We proposed a joint segmentation and classification system for hepatic lesions on US images based on user clicks. The system exhibits satisfactory performance in terms of segmentation (mean JI, 70.0% when trained with the binary classification and 68.5% when trained with the four-class classification) and classification (accuracy, 90.4% for the binary classification and 82.2% for the four-class classification). We expected that performing those two tasks jointly would improve the performance of each task compared to solving each task separately. While we decreased the execution time by combining the systems of each task, we also improved the performance for each task.

Hepatic tumor segmentation has long been an important topic, especially in the context of deep learning. Gruber et al developed a sequential network for liver and tumor segmentation in CT images, and a mean JI of 79.2% was reported for tumor segmentation [22]. Vorontsov et al reported per-lesion Dice similarity coefficients of 0.62 – 0.78 for segmentation of colorectal liver metastasis in CT images using a user-correction method. The Dice similarity coefficients were 0.14 – 0.68 without user correction [23]. Compared to the previous studies using CT images, our proposed system exhibited reasonable segmentation performance in US images (mean JI of 68.5 – 70.0%). Interestingly, while the method in [23] showed inaccurate segmentation for small lesions, the proposed system showed reasonable segmentation quality even for small lesions. This finding might be due to inherent differences between US and CT. In this study, we used two kinds of simulated user input, which were one-click and two-click mode. Two-click mode showed better segmentation performance (mean JI of 68.5%) compared with one-click mode (mean JI of 37.4%), especially for lesions with unclear margins, large size, and heterogeneous echogenicity. Considering the role of US in screening for malignancy such as HCC or metastasis, often demonstrating those features, two-click mode seems to be more suitable for clinical practice.

Most previous studies on hepatic tumor segmentation in US images were based on semiautomatic approaches due to the difficulty arising from speckles, shadows, and missing boundaries of US data [5]. Cvancarova et al used the active contour model or snakes to segment hepatic lesions in US images [24, 25]. In this model, segmentation is performed by transforming a snake, which is given by a user, to minimize the energy. When the energy is minimized, balance is achieved between the tension and rigidity of the snake and the degree of fitting to object boundaries. Egger et al proposed a graph-based semiautomatic approach, where the graph is constructed on a circular template placed by a user [26]. In this study, we adapted the semiautomatic segmentation method of [6] for US images, which requires a few user clicks. We used simulated user inputs that reflect different styles of real user input. Learning the user input together with an image enables the network to understand users’ intentions. Because the proposed method performs the segmentation by a trained end-to-end network and does not require complex optimization procedures, it works faster than the previous methods. While the execution times of the previous studies were a few seconds [25, 26], the mean execution time of our system was less than 0.1 s.

Although US is a widely used modality for screening hepatic lesions, it has limitations in terms of diagnostic accuracy of lesions due to its operator-dependent nature, and overlapping sonographic findings of solid hepatic lesions [27]. In clinical practice, US is considered a first-line modality that needs further examinations such as CT or MRI [28]. Human performance of differentiating benign and malignant focal hepatic lesions using only B-mode US images was reported to have high sensitivity (100%), but low specificity (18.9–30.2%) and AUC (0.665–0.706) [29]. Therefore, many studies have been conducted for hepatic lesion classification using deep learning. The system developed by Schmauch et al [30] reported a mean AUC of 0.916 in characterizing five types of hepatic lesions, including hemangioma, metastasis, HCC, cyst, and focal nodular hyperplasia. Hassan et al [31] developed a system classifying four types of focal hepatic lesions, including cyst, hemangioma, HCC, and normal liver parenchyma. The system exhibited good results in terms of accuracy (93.90 – 98.60%), sensitivity (95.70 – 98.30%), and specificity (92.60 – 98.90%). Our proposed system demonstrated higher specificity (86.0%) and AUROC (0.970), but slightly lower sensitivity (95.0%) in differentiating benign and malignant lesions than performance by radiologists in a previous study [29]. These results suggest that our proposed system could be used as a complementary tool for the radiologists. The AUC achieved by our system in characterizing focal hepatic lesions was 0.947, which was higher than the AUC obtained with the classification only system by Schmauch et al [30]. Our proposed system demonstrated slightly lower accuracy (82.2%), sensitivity (86.7%), and specificity (89.7%) compared with the performance of the system developed by Hassan et al [31]; however, we included many metastases cases with heterogeneous echogenicity and ill-defined or irregular borders, which were not included in the previous study by Hassan et al. Both previous studies reported relatively lower accuracy for solid lesions compared with cysts [30, 31]. This tendency is consistent with our results. The reduced accuracy for solid hepatic characterization could be related to overlapping and nonspecific US findings of those tumors. In our study, the classification performance of the joint system was higher than that of the classification only system in both binary and four-class classification tasks. We conjecture that the extracted features for the segmentation task, which is conducted jointly in the system, could help the classification task.

This study has several limitations. Although our dataset includes images obtained using diverse types of US machines, it does not include all different types of machines available in clinical practice; thus, the results may not be applied to US machines not included in this study. In addition, the relatively small numbers of metastases and HCC images in this study might affect the results.

In conclusion, the proposed joint segmentation and classification system for hepatic lesions in US images exhibited higher performance than the segmentation only and classification only systems. The proposed system could assist radiologists with minimal experience in US imaging by characterizing hepatic lesions.

## Supplementary information

ESM 1 (DOCX 28 kb)

## Abbreviations

AUROC: Area under the receiver operating characteristic curve
CNN: Convolutional neural network
FCN: Fully convolutional network
HCC: Hepatocellular carcinoma
JI: Jaccard index
ROI: Region-of-interest
US: Ultrasound

## Declarations

### Guarantor

The scientific guarantor of this publication is Jae Young Lee and Kyoung Mu Lee.

### Methodology

• Retrospective

• diagnostic or prognostic study

• performed at one institution
