# Supplementary material for: Joint segmentation and classification of hepatic lesions in ultrasound images using deep learning
Source: Eur Radiol. 2021 Apr 21;31(11):8733–42. doi: 10.1007/s00330-021-07850-9 (PMC8523410; doi:10.1007/s00330-021-07850-9)
Supplement: Supplementary file 1 — (DOCX 28 kb) [file 330_2021_7850_MOESM1_ESM.docx]

**Appendix 1. Mean and maximum number of pixels for each size category.**

| **Size** | size1 | size2 | size3 | size4 | size5 | size6 | size7 | size8 | size9 | size10 | **mean** |
| --- | --- | --- | --- | --- | --- | --- | --- | --- | --- | --- | --- |
| Cyst | 5.7x10^2^±  1.2x10^2^ (731) | 8.6x10^2^± 7.5x10 (988) | 1.1x10^3^± 9.3x10 (1308) | 1.5x10^3^± 1.0x10^2^ (1670) | 1.9x10^3^± 1.3x10^2^ (2107) | 2.4x10^3^± 2.1x10^2^ (2796) | 3.2x10^3^± 3.2x10^2^ (3865) | 4.8x10^3^± 6.2x10^2^ (6124) | 8.7x10^3^± 2.0x10^3^ (12656) | 3.9x10^4^± 3.2x10^4^ (196056) | 6.4x10^3^± 1.5x10^4^ |
| Hemangioma | 6.3x10^2^±  1.4x10^2^ (833) | 9.6x10^2^± 7.8x10 (1108) | 1.3x10^3^± 9.3x10 (1422) | 1.6x10^3^± 8.6x10 (1753) | 2.0x10^3^± 1.2x10^2^ (2169) | 2.4x10^3^± 1.3x10^2^ (2607) | 3.1x10^3^± 2.9x10^2^ (3608) | 4.3x10^3^± 4.4x10^2^ (5146) | 6.9x10^3^± 1.2x10^3^ (8886) | 2.7x10^4^± 2.7x10^4^ (140085) | 5.0x10^3^± 1.2x10^4^ |
| Metastasis | 1.7x10^3^± 4.5x10^2^ (2353) | 3.1x10^3^± 4.6x10^2^ (3976) | 4.6x10^3^±4.0x10^2^ (5332) | 6.3x10^3^± 6.0x10^2^ (7349) | 8.4x10^3^± 7.0x10^2^  (9655) | 1.1x10^4^± 8.6x10^2^ (12482) | 1.5x10^4^± 1.5x10^3^ (17449) | 2.1x10^4^± 2.1x10^3^ (24444) | 3.0x10^4^± 3.1x10^3^ (35403) | 6.2x10^4^± 2.7x10^4^ (162865) | 1.6x10^4^± 1.9x10^4^ |
| HCC | 1.1x10^3^± 2.3x10^2^ (1418) | 1.7x10^3^± 1.4x10^2^ (1940) | 2.2x10^3^± 1.6x10^2^ (2492) | 2.8x10^3^± 1.8x10^2^ (3167) | 3.6x10^3^± 3.0x10^2^ (4184) | 4.8x10^3^± 3.5x10^2^ (5384) | 6.2x10^3^± 5.5x10^2^ (7346) | 8.7x10^3^± 8.8x10^2^ (10452) | 1.4x10^4^± 2.8x10^3^ (19539) | 4.4x10^4^± 2.8x10^4^ (195363) | 9.0x10^3^± 1.9x10^4^ |
| **Mean** | 9.5x10^2^± 5.1x10^2^ | 1.6x10^3^± 9.3x10^2^ | 2.2 x10^3^± 1.4x10^3^ | 2.9 x10^3^± 2.0x10^3^ | 3.8x10^3^± 2.7x10^3^ | 4.9x10^3^± 3.5x10^3^ | 6.4x10^3^± 4.7x10^3^ | 9.2x10^3^± 6.7x10^3^ | 1.4x10^4^±9.2x10^3^ | 4.2x10^4^± 3.1x10^4^ | 8.8x10^3^± 1.5x10^4^ |

Data are demonstrated as mean ± standard deviation (maximum) number of pixels for each size category.

Note. HCC = hepatocellular carcinoma

**Appendix 2. Segmentation performance of the proposed systems with respect to size and types.**

| **Segm only (mean JI, %)** | size1 | size2 | size3 | size4 | size5 | size6 | size7 | size8 | size9 | size10 | **mean** |  |
| --- | --- | --- | --- | --- | --- | --- | --- | --- | --- | --- | --- | --- |
| Cyst | 58.7 | 68.9 | 68.5 | 72.3 | 73.6 | 72.7 | 77.6 | 75.0 | 79.7 | 79.3 | 72.6 |  |
| Hemangioma | 56.3 | 62.1 | 65.3 | 63.6 | 71.0 | 67.7 | 65.9 | 69.9 | 67.6 | 59.6 | 64.9 |  |
| Metastasis | 71.7 | 73.8 | 74.2 | 74.5 | 72.3 | 75.4 | 73.8 | 62.0 | 61.2 | 39.8 | 67.9 |  |
| HCC | 64.1 | 68.2 | 67.2 | 71.5 | 70.0 | 72.1 | 72.3 | 71.1 | 68.7 | 60.8 | 68.6 |  |
| **Mean** | 62.7 | 68.2 | 68.8 | 70.5 | 71.7 | 72.0 | 72.4 | 69.5 | 69.3 | 59.9 | 68.5 |  |
| **Joint (mean JI, %)** | size1 | size2 | size3 | size4 | size5 | size6 | size7 | size8 | size9 | size10 | **mean** | *p* |
| Cyst | 53.0 | 68.1 | 65.5 | 72.5 | 74.4 | 71.2 | 76.1 | 76.1 | 81.7 | 81.0 | 72.0 | 0.41 |
| Hemangioma | 59.8 | 65.1 | 62.9 | 67.8 | 70.0 | 69.8 | 67.6 | 66.1 | 67.3 | 51.5 | 64.8 | 0.93 |
| Metastasis | 71.8 | 69.4 | 74.5 | 70.7 | 73.8 | 71.5 | 73.4 | 62.6 | 69.1 | 45.0 | 68.2 | 0.71 |
| HCC | 61.9 | 59.5 | 68.4 | 70.0 | 69.1 | 70.9 | 76.5 | 70.8 | 72.1 | 70.0 | 68.9 | 0.72 |
| **Mean** | 61.6 | 65.5 | 67.8 | 70.2 | 71.8 | 70.9 | 73.4 | 68.9 | 72.5 | 61.8 | 68.5 | 0.95 |
| *P* | 0.57 | 0.08 | 0.40 | 0.83 | 0.93 | 0.30 | 0.40 | 0.61 | 0.04 | 0.29 |  |  |

Two-click user inputs were used and joint system trained with with four-class classification task.

Note. Segm only = segmentation only system, joint = joint segmentation and classification system, JI = Jaccard index, HCC = hepatocellular carcinoma

**Appendix 3. Classification performance of the proposed systems with respect to size and types.**

| **Cls only (accuracy, %)** | size1 | size2 | size3 | size4 | size5 | size6 | size7 | size8 | size9 | size10 | **mean** |  |
| --- | --- | --- | --- | --- | --- | --- | --- | --- | --- | --- | --- | --- |
| Cyst | 100 | 80.0 | 80.0 | 90.0 | 100 | 90.0 | 100 | 100 | 100 | 90.0 | 93.0 |  |
| Hemangioma | 100 | 100 | 100 | 100 | 100 | 70.0 | 100 | 50.0 | 80.0 | 80.0 | 88.0 |  |
| Metastasis | 40.0 | 70.0 | 70.0 | 70.0 | 90.0 | 70.0 | 100 | 60.0 | 60.0 | 70.0 | 70.0 |  |
| HCC | 70.0 | 80.0 | 70.0 | 80.0 | 80.0 | 60.0 | 70.0 | 70.0 | 60.0 | 40.0 | 68.0 |  |
| **Mean** | 77.5 | 82.5 | 80.0 | 85.0 | 92.5 | 72.5 | 92.5 | 70.0 | 75.0 | 70.0 | 79.8 |  |
| **Joint (accuracy, %)** | size1 | size2 | size3 | size4 | size5 | size6 | size7 | size8 | size9 | size10 | **mean** | *p* |
| Cyst | 100 | 90.0 | 70.0 | 100 | 100 | 90.0 | 100 | 100 | 94.0 | 100 | 94.4 | 0.36 |
| Hemangioma | 92.0 | 100 | 100 | 88.0 | 100 | 72.0 | 94.0 | 30.0 | 86.0 | 64.0 | 82.6 | <0.001 |
| Metastasis | 44.0 | 80.0 | 90.0 | 90.0 | 84.0 | 90.0 | 100 | 100 | 90.0 | 78.0 | 84.6 | <0.001 |
| HCC | 88.0 | 80.0 | 80.0 | 70.0 | 54.0 | 78.0 | 54.0 | 70.0 | 58.0 | 40.0 | 67.2 | 0.82 |
| **Mean** | 81.0 | 87.5 | 85.0 | 87.0 | 84.5 | 82.5 | 87.0 | 75.0 | 82.0 | 70.5 | 82.2 | 0.01 |
| *p* | 0.36 | 0.10 | 0.15 | 0.62 | 0.002 | <0.001 | 0.03 | 0.25 | 0.05 | 1.00 |  |  |

Two-click user inputs were used and both Cls only and joint system trained with four-class classification task.

Note. Cls only = Classification only system, joint = joint segmentation and classification system, HCC = hepatocellular carcinoma
